# Supplementary figures and images for: Cuproptosis genes in predicting the occurrence of allergic rhinitis and pharmacological treatment
Source: PLoS One. 2025 Feb 6;20(2):e0318511. doi: 10.1371/journal.pone.0318511 (PMC11801562; doi:10.1371/journal.pone.0318511)

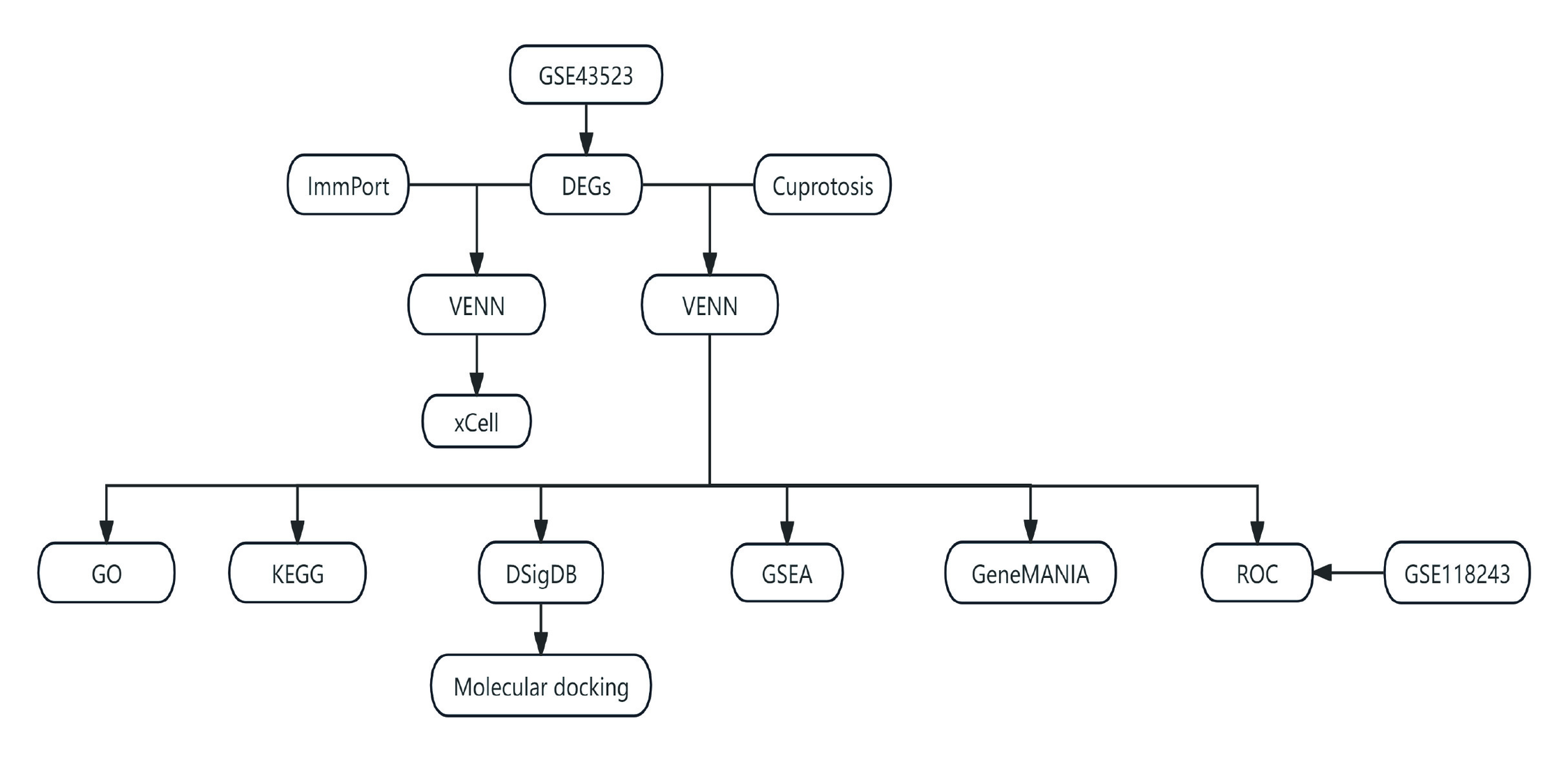

Supplement: S1 Fig — (TIF) [file pone.0318511.s001.tif]

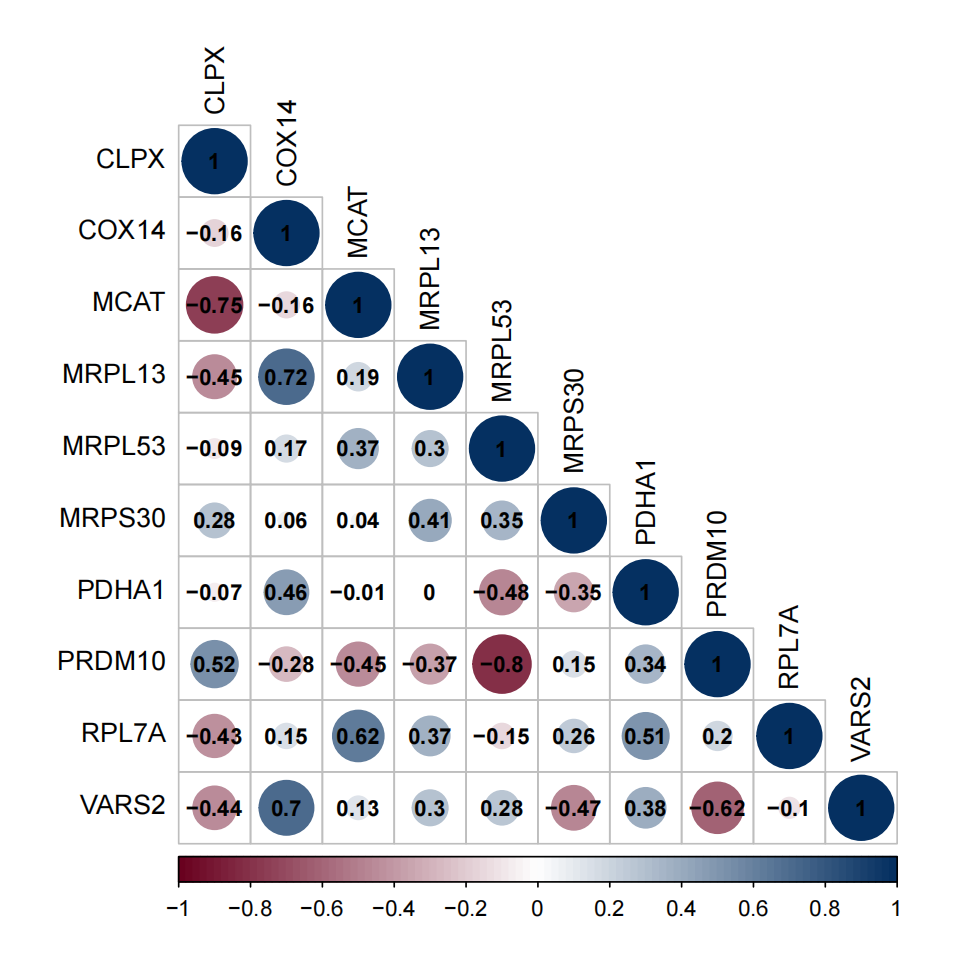

Supplement: S2 Fig — (TIF) [file pone.0318511.s002.tif]

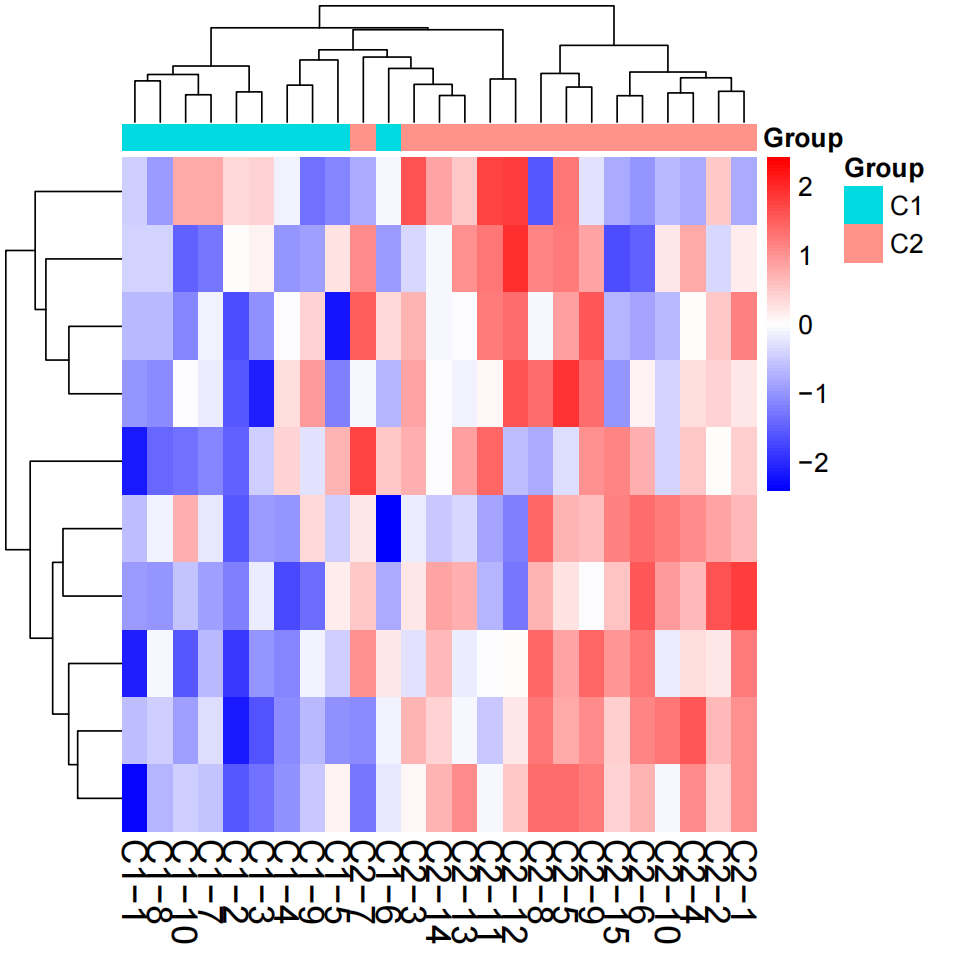

Supplement: S3 Fig — (TIF) [file pone.0318511.s003.tif]
