# Supplementary material for: Cuproptosis genes in predicting the occurrence of allergic rhinitis and pharmacological treatment
Source: PLoS One. 2025 Feb 6;20(2):e0318511. doi: 10.1371/journal.pone.0318511 (PMC11801562; doi:10.1371/journal.pone.0318511)
Supplement: Attachment — Submitted filename: Response to Reviewers.docx [file pone.0318511.s005.docx]

Dear Editor and Reviewers:

Thank you for your letter and the constructive comments on this article in your busy schedule. I have carefully read the comments that you have given, and have discussed and revised each of these issues. The following is my list of revisions. In addition, I have resubmitted a new manuscript in the revised state, with the revisions highlighted in red. If there are any incorrect answers or questions in the manuscript, please do not hesitate to let me know.I hope the revised manuscript will accepted for publication in the journal of Plos One.

Yours sincerely

Ti Yi

***Response to reviewers’ comments***

***Manuscript ID: PONE-D-23-34994***

***Title:Cuproptosis Genes in Predicting the Occurrence of Allergic Rhinitis and Pharmacological Treatment***

**Reviewer#1**

The structure of the paper was well structured. But it is having following corrections

1. Proofread the entire manuscript.

Response:I sincerely thank the reviewer for careful reading.As suggested by the reviewer,I have proofread the whole manuscript again.

2. Draw a graphical abstract to your study.

Response:I sincerely appreciate the valuable comments.I have added a graphical abstract in the Figure S1.

3. Compare your study with previous works.

Response:Thanks for your question. Cuproptosis is a newly discovered cell apoptosis mode, which has been confirmed to play an important role in the occurrence of nasopharyngeal carcinoma(PMID: 36618352), head and neck tumor(PMID: 36344660), breast cancer(PMID: 37918452), lung cancer(PMID: 37037146), and other malignant tumors, and is closely associated with inflammatory bowel disease(PMID: 37090740), Crohn's disease(PMID: 36466876), rheumatoid arthritis(PMID: 35990673), and other non-neoplastic diseases. However, no studies have confirmed the association between Cuproptosis and allergic rhinitis.

4. Explain the novelty of the proposed approach.

Response:Thanks for your question. Because there have no studies confirmed the association between Cuproptosis and allergic rhinitis before. This study investigates the potential association between SAR and cuproptosis molecular pathways by bioinformatic analyses of publicly available databases, to establish theoretical reference and guidance for the discovery and innovation of clinical treatment options for SAR.

**Reviewer#2**

Several major concerns have been addressed and should improve in the revised version.

1. The introduction section is too general. It should be objective-oriented and highlight what bioinformatics applications have already been accomplished, what updates are necessary, and what has been applied in the current article.

Response:Thanks for your question.In order to make the research methods more accessible to readers, I have added a graphical abstract in the Figure S1. Through bioinformatics methods, I not only analyzed the correlation between copper death related genes and allergic rhinitis, but also carried out drug prediction.
2. The data used in this article were published in 2015, and since then, many research groups have utilized it in their studies. However, those articles were not cited, and the results were not compared.

Response:Thank you for your careful reading. Previous studies have explored the pathogenesis of allergic rhinitis and other related studies on the data set published in 2015. This paper mainly discusses the correlation between allergic rhinitis and cuproptosis, and there is no obvious correlation between the two.

3. Author should provide the results such as DE analysis, pathway enrichment analysis and others in the supplementary table.

Response:Thanks for your question. I have submit the result of DE analysis, pathway enrichment analysis in the supplementary table.

4. The limitation mentioned, "Due to constraints in time and human resources, no further experimental verification has been conducted," is not satisfactory.

Response:Thank you for your suggestions. Further verification by experiment does make the whole paper more convincing. As a clinician who just graduated with a master's degree, I completed the statistical analysis and writing of this paper through my own efforts. Due to the lack of resources, I do not have chance for further experiments. Therefore, thank you again for your valuable advice.

5. Overall, the writing is of low quality. The sentence structure lacks consistency.

Response:Thanks for your suggestion.Before submission,I have already invited a friend who is native English speaker from the USA to help polish my article.

6. The sentence and logic should be corrected as follows:
"Differentially expressed genes (DEGs) between controls and SAR patients were analyzed using the limma package in R, as previously described [25]. DEGs were defined as genes with a fold change (FC) ≥ 1.3 and a p-value < 0.05. The dataset GSE118243 was employed as an independent validation set."
Additionally, the citation [25] in this context may be inappropriate, as it doesn't provide sufficient information about the implications of the limma package. The original limma package should be cited for clarity and accuracy.

Response:Thank you for the comments. We show the revisions in red font in the manuscript.

7. In the subsection "2.2 Cuproptosis and immune-related genes," the collection method for cuproptosis genes is mentioned, but crucial details, such as the rationale behind selecting this gene set and the objectives for its utilization, are missing.

Response:There are 347 cuprotosis related genes in the present study. We intersected cuprotosis related genes with differentially expressed genes through bioinformatics, VENN diagram showing the intersection of SAR differential genes and cuproptosis-related genes which is defined as the SAR/cuproptosis gene set. (D) Correlation analysis of the overlapping SAR/cuproptosis genes. The SAR/cuproptosis genes were analyzed with GO terms for major biological processes (E)

8. Figure 1D: A heatmap illustrating the correlation would be clearer than a circus. The author mentions, "Correlation analysis showed a strong correlation between these 10 SAR/cuprotosis genes." However, the importance of this correlation analysis and the implications of the results are not discussed and should be addressed.

Response:Thanks for your question. Although the correlation coefficient matrix heat map is very intuitive, it can look very laborious and take up a lot of space. The use of circular form to display the correlation coefficient matrix makes reader feel soft and rich. Through using the pearson correlation coefficient,Its value is between -1 and 1, between 0 and 1is correlated and we use the red color.

9. In the results section, it is reported that there are 10 overlapped genes between the 478 DEGs and the 347 cuproptosis genes. However, the statistical significance level of this overlap is not reported.

Response:Thanks for your question. These ten SAR/cuprotosis-related genes include seven upregulated (VARS2, MCAT, RPL7A, MRPL13, PDHA1, MRPL53, COX14) and three downregulated genes (MRPS30, PRDM10, CLPX).The possible correlations between the SAR/cuprotosis-related genes is displayed on figureS2.

10. In the result section, only the terms of the pathways are reported for the GO and KEGG pathway analysis. However, there is a lack of discussion regarding the association of these pathways with Allergic Rhinitis.

Response:Thanks for your question. This pathways is important for Gene expression and transcription and I will further verify it in the later stage If the circumstances permit.

11. Lines 265-276: The gene MRPS30 is primarily associated with breast cancer or tumors, with no direct relation to Allergic Rhinitis. The author attempts to establish a distant connection, which may be challenging to substantiate experimentally. The same comment applies to the other three signature genes.

Response:Thank you for your suggestions. Further verification by experiment does make the whole paper more convincing. As a clinician who just graduated with a master's degree, I completed the statistical analysis and writing of this paper through my own efforts. Due to the lack of resources, I do not have chance for further experiments.

**Reviewer #3:**

• Many figures suffer from poor quality, appear blurry and difficult to read, and need to change with high resolution ones.

Response:After I get the analysis results through R , I use the app“ AI” to combine them into graphs, and I have tried to make the pictures and figures clear to ensure readers' reading.

• The molecular docking results should be presented and discussed with clear justifications for the selection of 1,5-isoquinolinediol and gefitinib as potential SAR treatments, considering factors like binding affinity and specificity.

Response:Thank you for your suggestions.In line 250-264,the associated cytokine responses of SAR I described are common in allergic reactions.Thank you for your detailed reading.

• There is no apparent connection between the discussed paragraphs (line numbers 250-264) and the context of SAR and associated cytokine responses. There is a noticeable gap, and the authors have not adequately addressed the association of these responses with the identified genes. Proper transition is needed to bridge the gap between SAR, cytokines response and identified genes.

Response:Thank you for your suggestions.In line 250-264,the associated cytokine responses of SAR I described are common in allergic reactions.Thank you for your detailed reading.

• It is advised that the authors should define abbreviations at least in the first instance. E.g. GEO in abstract, please check for other in entire manuscript.

Response:Thank you for the comments. We show the revisions in red font in the manuscript.

• There are a lot of definite and indefinite articles missing along with some grammatical errors, also please correct the manuscript for undefined spaces.

Response:Thanks for your suggestion.Before submission,I have already invited a friend who is native English speaker from the USA to help polish my article.

• Number of spelling mistakes/typo errors.

Response:I feel sorry for my carelessness.In my resubmitted manuscript,the type has been revised.Thanks for your correction.

• Citations and references must be in the correct and uniform format, Italics missing in some places.

Response:Thanks for your careful checks.I borrowed the format of some paper previously published in the journal of “Plos One” and used the “Vancouver” style in reference and some fonts have been corrected to italics.Thanks for your correction.
