# Supplementary material for: Cuproptosis genes in predicting the occurrence of allergic rhinitis and pharmacological treatment
Source: PLoS One. 2025 Feb 6;20(2):e0318511. doi: 10.1371/journal.pone.0318511 (PMC11801562; doi:10.1371/journal.pone.0318511)
Supplement: Attachment — Submitted filename: Response to Reviewers.docx [file pone.0318511.s006.docx]

Dear Editor and Reviewers:

Thank you for your letter and the constructive comments on this article in your busy schedule. I have carefully read the comments that you have given, and have discussed and revised each of these issues. The following is my list of revisions. In addition, I have resubmitted a new manuscript in the revised state, with the revisions highlighted in red. If there are any incorrect answers or questions in the manuscript, please do not hesitate to let me know.I hope the revised manuscript will accepted for publication in the journal of Plos One.

Yours sincerely

Ti Yi

***Response to reviewers’ comments***

***Manuscript ID: PONE-D-23-34994***

***Title:Cuproptosis Genes in Predicting the Occurrence of Allergic Rhinitis and Pharmacological Treatment***

Please use the space provided to explain your answers to the questions above. You may also include additional comments for the author, including concerns about dual publication, research ethics, or publication ethics. (Please upload your review as an attachment if it exceeds 20,000 characters)

**Reviewer#2**

I and reviewer 3 suggested improvements to the writing. In response, the author stated, "Before submission, I had already enlisted the help of a friend who is a native English speaker from the USA to assist in polishing my article."However, having a native speaker from a different field revise a research article may not be sufficient.Additionally, the discussion of the results is inadequate. I recommend discussing the GO and KEGG pathway enrichment results to establish connections between the disease and the identified pathways. I did not ask an experimental study for the GO and KEGG pathway.The author's overall response likely avoids the reviewer's query

Response: I sincerely appreciate the valuable comments. Your valuable advice has inspired me, I've added GO and KEGG results in the discussion section, re-checked the full text, and made corresponding modifications.

**Reviewer #3**:

The authors have effectively considered and incorporated the concerns highlighted earlier. The revisions made by the authors have substantially improved the manuscript. I think, the modified version now appears well-prepared for publication.

Response: Thanks for your affirmation. Your previous suggestions have given me great inspiration.
